# Supplementary figures and images for: Discovery of novel benzophenone integrated derivatives as anti-Alzheimer’s agents targeting presenilin-1 and presenilin-2 inhibition: A computational approach
Source: PLoS One. 2022 Apr 8;17(4):e0265022. doi: 10.1371/journal.pone.0265022 (PMC8993008; doi:10.1371/journal.pone.0265022)

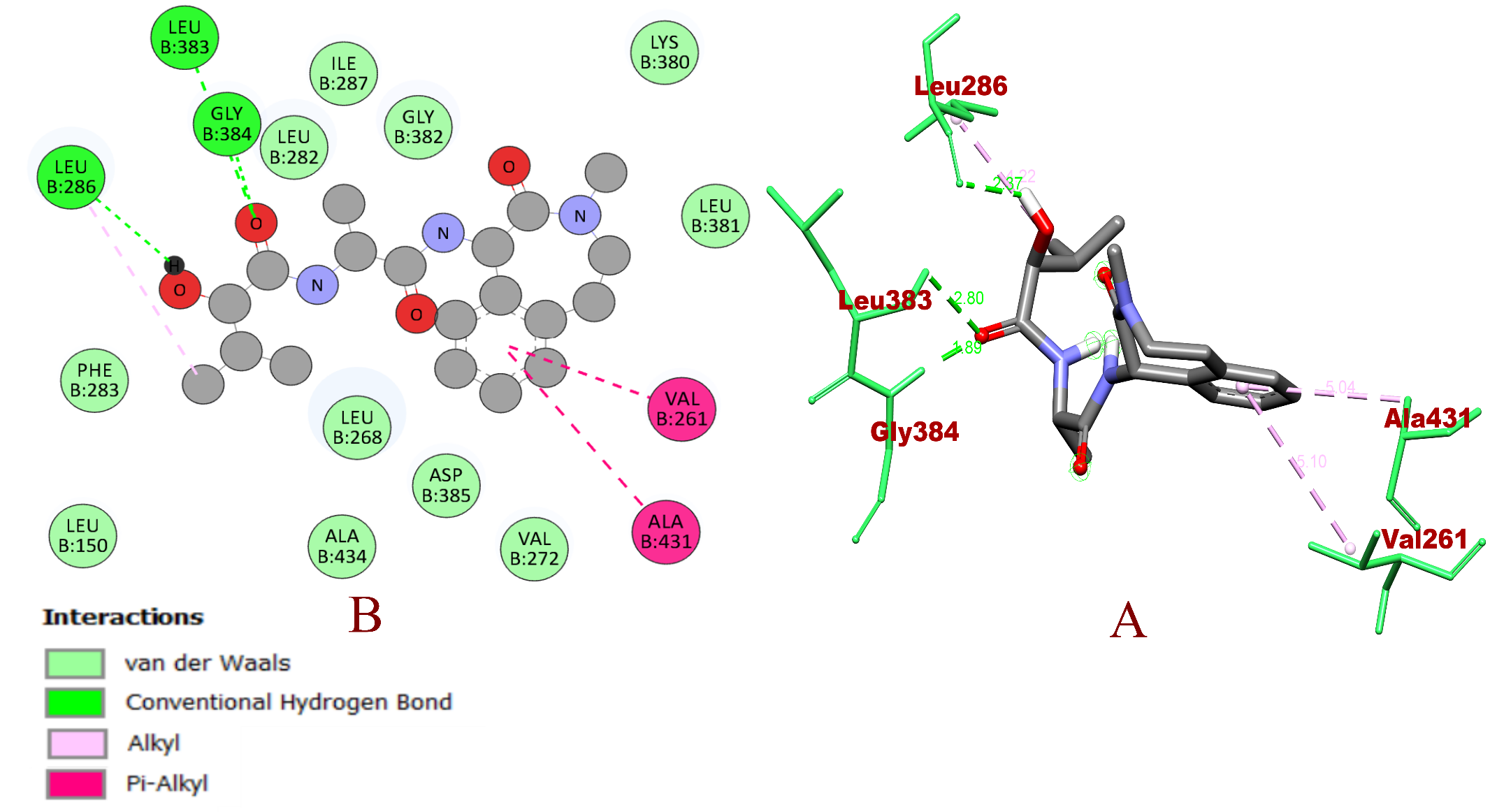


**S1 Fig. Visualization of binding interactions of LY450139 with PSEN-1.**

Supplement: S1 Fig — (DOCX) [file pone.0265022.s001.docx]

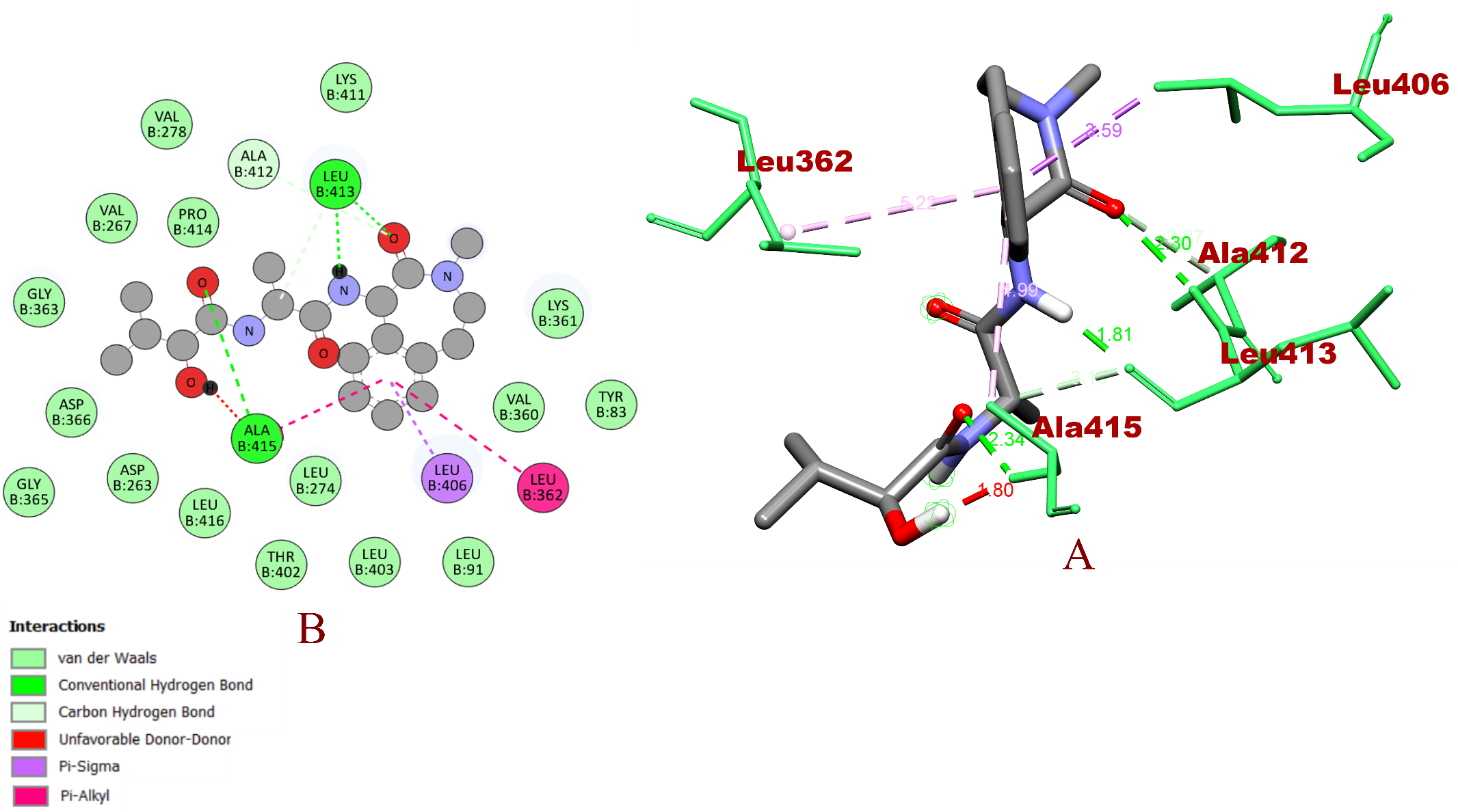


**S2 Fig. Visualization of binding interactions of LY450139 with PSEN-2.**

Supplement: S2 Fig — (DOCX) [file pone.0265022.s002.docx]
