# Supplementary material for: Discovery of novel benzophenone integrated derivatives as anti-Alzheimer’s agents targeting presenilin-1 and presenilin-2 inhibition: A computational approach
Source: PLoS One. 2022 Apr 8;17(4):e0265022. doi: 10.1371/journal.pone.0265022 (PMC8993008; doi:10.1371/journal.pone.0265022)
Supplement: S1 Table — (DOCX) [file pone.0265022.s003.docx]

**S1 Table. Structural differences and results of molecular docking simulation of BID’s with PSEN-2 and PSEN-2 proteins.**

| **Sl. No** | **Structure** | **Molecular structural**  **differences** | **Docking with PSEN-1** | | | **Docking with PSEN-2** | | |
| --- | --- | --- | --- | --- | --- | --- | --- | --- |
|  |  |  | **BA** | **NB** | **HB** | **BA** | **NB** | **HB** |
| 1 |   2-((4-Benzoyl-2-methylphenoxy) methyl)-5-(1-methylindole-3-yl)-1,3,4-oxadiazole (BID-1) | Basic skeleton comprises of N-methyl indole and 2-Methyl benzophenone bridged via 1,3,4-oxadiazole,  No additional substituents at benzophenone ring | -8.4 | 13 | 4 | -8.6 | 14 | 2 |
| 2 |   2-([4-(2-Chlorobenzoyl)-2-methylphenoxy] methyl)-5-(1-methylindole-3-yl)-1,3,4-oxadiazole (BID-2) | With basic skeleton, Chloro group is present at ortho position of benzoyl ring of benzophenone | -8.6 | 10 | 3 | -8.4 | 11 | 4 |
| 3 |   2[4(4-methylbenzoyl)-2-methylphenoxy]-5-N-methyl-indole 1,3,4-oxadiazoles (BID-3) | With basic skeleton, methyl group is present at para position of benzoyl ring of benzophenone | -8.5 | 12 | 4 | -8.8 | 14 | 5 |
| 4 |   2[4(4-methoxybenzoyl)-2-methylphenoxy]-5-N-methyl-indole 1,3,4-oxadiazoles (BID-4) | With basic skeleton, Methoxy group is present at para position of benzoyl ring of benzophenone | -9.1 | 12 | 5 | -9.2 | 14 | 2 |
| 5 |   2[4(3-bromobenzoyl)-2-methylphenoxy]-5-N-methyl-indole 1,3,4-oxadiazoles (BID-5) | With basic skeleton, bromo group is present at meta position of benzoyl ring of benzophenone | -8.7 | 8 | 1 | -8.5 | 11 | 1 |
| 6 |   2[4(4-fluorobenzoyl)-2-methylphenoxy]-5-N-methyl-indole 1,3,4-oxadiazoles (BID-6) | With basic skeleton, fluoro group is present at para position of benzoyl ring of benzophenone | -9.4 | 13 | 2 | -9.0 | 6 | - |
| 7 |   N-methyl-1H-indole-3-carboxylic acid N'-[2-(4-benzoyl-2-methyl-phenoxy)-acetyl]-hydrazide (BID-7) | Basic skeleton comprises of N-methyl indole and 2-Methyl benzophenone bridged via amide linkage,  No additional substituents at benzophenone ring | -9.1 | 13 | 5 | -8.7 | 9 | 1 |
| 8 |   N-methyl-1H-indole-3-carboxylic acid N'-[2-(4-(2-chloro)-benzoyl-2-methyl-phenoxy)-acetyl]-hydrazide (BID-8) | With basic skeleton, Chloro group is present at ortho position of benzoyl ring of benzophenone | -9.8 | 9 | 5 | -8.7 | 13 | - |
| 9 |   N-methyl-1H-indole-3-carboxylic acid N'-[2-(4-(3-chloro)-benzoyl-2-methyl-phenoxy)-acetyl]-hydrazide (BID-9) | With basic skeleton, chloro group is present at meta position of benzoyl ring of benzophenone | -8.5 | 12 | 2 | -8.1 | 15 | 4 |
| 10 |   N-methyl-1H-indole-3-carboxylic acid N'-[2-(4-(2-bromo)-benzoyl-2-methyl-phenoxy)-acetyl]-hydrazide (BID-10) | With basic skeleton, bromo group is present at ortho position of benzoyl ring of benzophenone | -9.9 | 8 | 5 | -8.8 | 14 | 3 |
| 11 |   N-methyl-1H-indole-3-carboxylic acid N'-[2-(4-(4-bromo)-benzoyl-2-methyl-phenoxy)-acetyl]-hydrazide (BID-11) | With basic skeleton, bromo group is present at para position of benzoyl ring of benzophenone | -10.0 | 11 | 5 | -8.7 | 14 | 3 |
| 12 |   N-methyl-1H-indole-3-carboxylic acid N'-[2-(4-(4-fluro)-benzoyl-2-methyl-phenoxy)-acetyl]-hydrazide (BID-12) | With basic skeleton, fluoro group is present at para position of benzoyl ring of benzophenone | -9.3 | 10 | 5 | -9.0 | 12 | 2 |
| 13 |   N-methyl-1H-indole-3-carboxylic acid N'-[2-(4-(4-methoxy)-benzoyl-2-methyl-phenoxy)-acetyl]-hydrazide (BID-13) | With basic skeleton, methoxy group is present at para position of benzoyl ring of benzophenone | -9.2 | 12 | 8 | -8.3 | 12 | 3 |
| 14 |   N-methyl-1H-indole-3-carboxylic acid N'-[2-(4-(4-methyl)-benzoyl-2-methyl-phenoxy)-acetyl]-hydrazide (BID-14) | With basic skeleton, methyl group is present at para position of benzoyl ring of benzophenone | -7.8 | 10 | 2 | -9.0 | 8 | 1 |
| 15 |   N-methyl-1H-indole-3-carboxylic acid N'-[2-(4-(2-methyl)-benzoyl-2-methyl-phenoxy)-acetyl]-hydrazide (BID-15) | With basic skeleton, methyl group is present at ortho position of benzoyl ring of benzophenone | -9.5 | 11 | 5 | -9.1 | 10 | 2 |
| 16 | ****  **N-methyl-1H-indole-3-carboxylic acid N'-[2-(4-(3-methyl)-benzoyl-2-methyl-phenoxy)-acetyl]-hydrazide (BID-16)** | **With basic skeleton, methyl group is present at meta position of benzoyl ring of benzophenone also it contains another methyl group at ortho position of phenyl ring** | **-10.2** | **12** | **4** | **-9.4** | **15** | **2** |
| 17 |   (5-bromo-N-methyl indol-3-yl)-acetic acid 4-(4-methoxy)-benzoyl-2-methyl phenyl ester (BID-17) | Basic skeleton comprises of 5-bromo N-methyl indole and 2-Methyl benzophenone bridged via phenyl ester linkage.  No additional substituents at benzophenone ring | -7.8 | 14 | 2 | -7.8 | 7 | - |
| 18 |   (5-bromo-N-methyl indol-3-yl)-acetic acid 4-(6-methyl)-benzoyl-2-methyl phenyl ester (BID-18) | With basic skeleton, methyl group is present at ortho position of benzoyl ring of benzophenone | -8.1 | 17 | 2 | -7.9 | 7 | 1 |
| 19 |   (5-bromo-N-methyl indol-3-yl)-acetic acid 4-(5-chloro)-benzoyl-2-methyl phenyl ester (BID-19) | With basic skeleton, chloro group is present at meta position of benzoyl ring of benzophenone | -7.6 | 9 | 1 | -8.1 | 12 | 2 |
| 20 |   (5-bromo-N-methyl indol-3-yl)-acetic acid 4-(4-bromo)-benzoyl-2-methyl phenyl ester (BID-20) | With basic skeleton, bromo group is present at para position of benzoyl ring of benzophenone | -7.8 | 8 | 1 | -8.2 | 3 | - |
| 21 |   (5-bromo-N-methyl indol-3-yl)-acetic acid 4-(4-chloro)-benzoyl-2-methyl phenyl ester (BID-21) | With basic skeleton, 2 chloro groups were present at ortho and para position of benzoyl ring of benzophenone | -7.9 | 5 | 2 | -8.4 | 12 | 4 |
| 22 |   (5-bromo-N-methyl indol-3-yl)-acetic acid 4-(4-methyl)-benzoyl-2-methyl phenyl ester (BID-22) | With basic skeleton, methyl group is present at para position of benzoyl ring of benzophenone | -7.7 | 12 | 1 | -8.0 | 8 | - |
| 23 |   (5-bromo-N-methyl indol-3-yl)-acetic acid 4-(5-methyl)-benzoyl-2-methyl phenyl ester (BID-23) | With basic skeleton, methyl group is present at meta position of benzoyl ring of benzophenone | -7.9 | 12 | - | -8.1 | 7 | - |
| 24 |   (5-bromo-N-methyl indol-3-yl)-acetic acid 4-(6-bromo)-benzoyl-2-methyl phenyl ester (BID-24) | With basic skeleton, bromo group is present at ortho position of benzoyl ring of benzophenone | -8.4 | 13 | 3 | -8.3 | 9 | 2 |
| 25 |   (5-bromo-N-methyl indol-3-yl)-acetic acid 4-(4-fluro)-benzoyl-2-methyl phenyl ester (BID-25) | With basic skeleton, fluoro group is present at para position of benzoyl ring of benzophenone | -8.3 | 14 | 4 | -8.6 | 13 | 4 |
| 26 |   (5-bromo-N-methyl indol-3-yl)-acetic acid 4-benzoyl-2-methyl phenyl ester (BID-26) | Basic skeleton comprises of 5-bromo N-methyl indole and 2-Methyl benzophenone bridged via phenyl ester linkage.  No additional substituents at benzophenone ring | -7.7 | 8 | 1 | -8.0 | 12 | 2 |
| 27 |   [4-(1H-benzimidazol-2-ylmethoxy)-3-methyl-phenyl]-phenyl-methanone (BID-27) | Basic skeleton comprises of benzimidazole, and 2-Methyl benzophenone bridged via phenoxy linkage.  No additional substituents at benzophenone ring | -8.1 | 12 | 3 | -8.5 | 8 | 1 |
| 28 |   [4-(1H-benzimidazol-2-ylmethoxy)-3-methyl-phenyl]-(2-fluoro-phenyl)-methanone (BID-28) | With basic skeleton, fluoro group is present at ortho position of benzoyl ring of benzophenone | -8.3 | 9 | 2 | -8.6 | 10 | 1 |
| 29 |   [4-(1H-benzimidazol-2-ylmethoxy)-3-methyl-phenyl]-(4-fluoro-phenyl)-methanone (BID-29) | With basic skeleton, fluoro group is present at para position of benzoyl ring of benzophenone | -8.4 | 8 | 1 | -8.7 | 9 | 1 |
| 30 |   [4-(1H-benzimidazol-2-ylmethoxy)-3-methyl-phenyl]-(3-bromo-phenyl)-methanone (BID-30) | With basic skeleton, bromo group is present at meta position of benzoyl ring of benzophenone | -8.3 | 12 | 3 | -8.8 | 11 | 1 |
| 31 |   [4-(1H-benzimidazol-2-ylmethoxy)-3-methyl-phenyl]-(3-fluoro-phenyl)-methanone (BID-31) | With basic skeleton, fluoro group is present at meta position of benzoyl ring of benzophenone | -8.1 | 13 | 2 | -8.9 | 9 | 1 |
| 32 |   [4-(1H-benzimidazol-2-ylmethoxy)-3-methyl-phenyl]-(4-methoxy-phenyl)-methanone (BID-32) | With basic skeleton, methoxy group is present at para position of benzoyl ring of benzophenone | -8.1 | 10 | 6 | -8.3 | 10 | 1 |
| 33 |   [4-(1H-benzimidazol-2-ylmethoxy)-3-methyl-phenyl]-(2-chloro-phenyl)-methanone (BID-33) | With basic skeleton, chloro group is present at ortho position of benzoyl ring of benzophenone | -8.2 | 7 | 1 | -8.6 | 9 | 1 |
| 34 |   [4-(1H-benzimidazol-2-ylmethoxy)-3-methyl-phenyl]-o-tolyl-methanone (BID-34) | With basic skeleton, methyl group is present at ortho position of benzoyl ring of benzophenone | -8.0 | 8 | 1 | -8.4 | 8 | 1 |
| 35 |   [4-(1H-benzimidazol-2-ylmethoxy)-3-methyl-phenyl]-m-tolyl-methanone (BID-35) | With basic skeleton, ortho group is present at meta position of benzoyl ring of benzophenone | -8.5 | 10 | 2 | -8.8 | 11 | 1 |
| 36 |   [4-(1H-benzimidazol-2-ylmethoxy)-3-methyl-phenyl]-(4-bromo-phenyl)-methanone (BID-36 ) | With basic skeleton, bromo group is present at para position of benzoyl ring of benzophenone | -8.1 | 9 | - | -8.2 | 10 | 1 |
| 37 |   [4-(1H-benzimidazol-2-ylmethoxy)-3,5-dimethyl-phenyl]-phenyl-methanone (BID-37) | With basic skeleton, 2 methyl groups were present at ortho position of phenyl ring of benzophenone | -8.1 | 7 | 2 | -8.7 | 9 | 1 |
| 38 |   [4-(1H-benzimidazol-2-ylmethoxy)-3,5-dimethyl-phenyl]-p-tolyl-methanone (BID-38) | With basic skeleton, 2 methyl groups were present at ortho position of phenyl ring and 1 methyl group is present at para position of benzoyl ring of benzophenone | -8.5 | 13 | 3 | -8.9 | 12 | 1 |
| 39 |   [4-(1H-benzimidazol-2-ylmethoxy)-3,5-dimethyl-phenyl]-(4-fluoro-phenyl)methanone (BID-39) | With basic skeleton, 2 methyl groups were present at ortho position of phenyl ring and fluoro group is present at para position of benzoyl ring of benzophenone | -8.4 | 12 | 4 | -8.9 | 9 | 1 |
| 40 |   [4-(1H-benzimidazol-2-ylmethoxy)-3,5-dimethyl-phenyl]-(4-methoxy-phenyl) methanone (BID-40) | With basic skeleton, 2 methyl groups were present at ortho position of phenyl ring and 1 methoxy group is present at para position of benzoyl ring of benzophenone | -8.2 | 10 | 1 | -8.5 | 11 | 1 |
