# Supplementary material for: Discovery of novel benzophenone integrated derivatives as anti-Alzheimer’s agents targeting presenilin-1 and presenilin-2 inhibition: A computational approach
Source: PLoS One. 2022 Apr 8;17(4):e0265022. doi: 10.1371/journal.pone.0265022 (PMC8993008; doi:10.1371/journal.pone.0265022)
Supplement: S2 Table — (DOCX) [file pone.0265022.s004.docx]

**S2 Table. Binding affinity and interactions of LY450139 with PSEN-1 and PSEN-2.**

| **Sl. No.** | **Name of the compound** | **Binding affinity**  **(kcal/mol)** | **Hydrogen bonds** | **Hydrophobic bonds** | | |
| --- | --- | --- | --- | --- | --- | --- |
|  |  |  |  | **Pi-Sigma** | **Alkyl** | **Pi-alkyl** |
| 1 | PSEN-1 with LY450139 | -7.6 | LEU B: 383 ( 2.79), GLY B: 384 ( 1.88), LEU B: 286 ( 2.36) | - | LEU B: 286 (4.21) | VAL B: 261 (5.09), ALA B: 431 ( 5.03) |
| 2 | PSEN-2 with LY450139 | -7.6 | LEU B: 413 ( 2.30), ALA B: 415 ( 2.33), LEU B: 413 ( 1.80), ALA B: 412 ( 3.26), LEU B: 413 ( 3.14) | LEU B: 406 (3.59) | - | LEU B: 362 ( 5.21), ALA B: 415 ( 4.98) |
