# Supplementary material for: Discovery of novel benzophenone integrated derivatives as anti-Alzheimer’s agents targeting presenilin-1 and presenilin-2 inhibition: A computational approach
Source: PLoS One. 2022 Apr 8;17(4):e0265022. doi: 10.1371/journal.pone.0265022 (PMC8993008; doi:10.1371/journal.pone.0265022)
Supplement: Attachment — Submitted filename: Author Response (1).docx [file pone.0265022.s005.docx]

**Response to the Reviewer’s comments**

**Dear Professor**

All the authors sincerely thank the editor/s and the reviewers for considering our manuscript significant and providing their valuable time and suggestion to improve the quality of the manuscript. Considering the thoughtful inputs the entire manuscript has been reformed after carrying out necessary incorporations. All the changes incorporated by the authors have been carried using “**TRACK CHANGES**”. The changes have been followed and remodelled in the respective sections as per the instructions given by the reviewers and as per the guidelines of the journal. The authors have also provided the additional file without track changes (Final revised file). Also we wish to add a supplementary data (supportive data) for our studies and the same has been provided while submission.

Thank you for your support and your hard work. I am herewith requesting your kind self to add Few authors (**JAYANTHI M K ^b^, ASHWINI P ^c^, LAKSHMI V RANGANATHA ^d^,** [**SHAUKATH ARA KHANUM**](https://www.ncbi.nlm.nih.gov/pubmed/?term=Khanum%20SA%5BAuthor%5D&cauthor=true&cauthor_uid=34092960) **^e^, EKATERINA SILINA ^f^,** **VICTOR STUPIN ^g^, RAGHU RAM ACHAR ^h^**) with their affiliations (^b^ Department of Pharmacology, JSS Medical College, JSS Academy of Higher Education and Research, Mysuru-570015, Karnataka, India; ^c^ Department of Microbiology, School of Life Sciences, JSS Academy of Higher Education and Research, Mysuru-570015, Karnataka, India; ^d^ Department of Chemistry, The National Institute of Engineering, Manandavadi Road, Mysuru 570008, Karnataka, India; ^e^ Department of Chemistry, Yuvaraja's College (Autonomous), University of Mysore, Mysuru - 570 005, Karnataka, India; ^f^ Department of Human Pathology, I.M. Sechenov First Moscow State Medical University (Sechenov University). Trubetskaya St., 8, Moscow, 119991, Russia; ^g^ Department of Hospital Surgery №1, N.I. Pirogov Russian National Research Medical University (RNRMU), Ostrovityanova St. 1, Moscow, 119991, Russia; ^h^ Division of Biochemistry, School of Life Sciences, JSS Academy of Higher Education and Research, Mysuru-570015, Karnataka, India) to the manuscript. All the authors have contributed their valuable inputs and revised critically to make this manuscript in a good form. In this regard, we hereby request you to add them and their affiliations to the upcoming manuscript. The final author orders are **Reshma Mary Martiz, Shashank M Patil, Ramith Ramu^*^, Jayanthi M K, Ashwini P, Lakshmi V Ranganatha,** [**Shaukath Ara Khanum**](https://www.ncbi.nlm.nih.gov/pubmed/?term=Khanum%20SA%5BAuthor%5D&cauthor=true&cauthor_uid=34092960)**, Ekaterina Silina,** **Victor Stupin, Raghu Ram Achar.**

Also, while submitting the initial manuscript to the journal, the article was communicated with THREE authors and the remaining authors had not given their consent since they were out of station and now they have given their consent. In this regard, we request your kind self to add the remaining authors. All the authors agree and affirm to the changes made in terms of author order and inclusion of co-authors. Also, we hereby state that we do not have any conflicts of interest for the same.

Accordingly, the issues that are raised by reviewers are being addressed in the revised manuscript and here we answer it point-by-point:

**Reviewer 1 comments**

The study analysed the binding affinity between benzophenones and PSEN-1/2 for demonstrating the pharmacotherapeutic potential by inhibiting PSEN-1/2. However, the study only focused on analysing the affinities and interactions, without further investigation of the preferential binding pocket for the inhibition effect. Herein, it is hard to conclude the inhibition efficiency of screened benzophenone from these preliminary data, and many issues need to be addressed as followings:

1. Figure 3-4, the binding pocket and binding sequence labelled in different colours should be clearly explained in the figure caption.

Response: As per the reviewer’s suggestion, binding pocket and binding sequence labelled in different colours (for both PSEN-1 and PSEN-2) have been explained in figure captions of Figure 3 (line 201-204) and Figure 4 (line 206-209).

2. Table 3-4, the in parallel comparison of binding affinity and non-bonding interactions of various BIDs are encouraged to present in column figures for better illustration. Also, please highlight the molecule structural differences of these BIDs in different colour.

Response: As per the reviewer’s suggestion, Table 3 and Table 4 have been merged. The merged table (Table 3, line 225) now contains the structural differences of the BIDs highlighted in different colour. Also, binding affinity, total number of non-bonding interactions, total number of hydrogen bonds formed by BIDs during the docking simulation have also been given in adjacent columns. For the better illustration of these interaction parameters, Figure 5 and Figure 6 have been added, which represent the different docking parameters of PSEN-1 and PSEN-2, respectively.

3. Figure 5-6, it is hard to distinguish between the Pi-alkyl and alkyl interactions.

Response: As per the reviewer’s suggestion, colour of the Pi-alkyl interactions have been changed to dark pink colour (now Figure 7 and Figure 8). Also, row headings have been added in Table 4, which clearly describes the types of interactions to avoid confusion.

4. The binding interactions between BIDs and PSEN-1/2 were analysed in silico, but for the inhibition effect, how this binding contributes to the inhibition? Whether by stabilize the PSEN-1/2 conformation or block the active sites? All these need to be further introduced to demonstrate the inhibition benefits.

Response: As per the reviewer’s suggestion, in silico interaction of BIDs have now been supported with suitable information in “Discussion” section (lines 439-462) and supplementary data. In order to do this, authors have carefully performed the binding pocket analysis of both PSEN proteins, followed by the interaction analysis of BIDs with specific amino acid residues. We have identified a few key amino acid residues from the literature analysis, binding to which induces the inhibition activity. Binding interactions of BIDs also reveal that the key amino acids have been bound with the BIDs. Therefore, the interaction is oriented towards the inhibition of the protein activity by blocking the active binding residues.

From previous studies conducted on γ-secretase, which consists of PSEN-1 and PSEN-2 proteins as functional subunits, we have provided the information of in silico inhibition of γ-secretase. The same inhibitor compound (LY450139) used in the study was docked with our homology model built PSEN-1 and PSEN-2 proteins. This has produced similar binding interactions like BIDs, which depicts that the binding interactions of BIDs have been oriented towards the inhibition, not the activation of the target proteins.

5. In addition to the binding affinity, the binding pocket should also be analysed for screening the best pose of BID-16 with PSEN-1/2.

Response: As per the reviewer’s suggestion, a thorough analysis of binding pockets of both PSEN-1 and PSEN-2 was conducted using the literature given in the UniProt database website. The literature survey resulted in the identification of few key amino residues, binding to which causes the inhibition of the protein activity. Results from our molecular docking studies reveal that BID-16 binds to the same amino acid residues, which shows that BID-16 as a potential inhibitor of both the PSEN-1 and PSEN-2 proteins. Difference in MD simulation plots were also given to highlight the impact of interaction (lines 476-481). During the virtual screening of the different binding poses, the first binding pose with zero RMSD and most negative binding affinity was selected. The same binding pose was found to interact with the key residues, in comparison with the other 9 binding poses.

**Reviewer 2 comments**

In the manuscript PONE-D-21-34824, the study evaluates the interaction of BIDs through molecular docking simulations, molecular dynamics simulations, and binding free energy calculations. Combinations of these approaches are acceptable. Although the manuscript is informative, there are still some questions that need to be addressed (see comments listed below).

1. The false-positive rate of molecular docking is very high in the absence of experimental support. The literature listed by the author only mentions the inhibition of AChE/BACE-1 by Benzophenones, not PSEN-1/PSEN-2. Therefore, there is little significance in performing molecular docking study in such cases.

Response: As per the reviewer’s suggestion, we conducted an extensive literature survey on the involvement of BIDs in the induction of anti-Alzheimer’s activity. However, there have been no studies performed *in vitro* for the inhibition of PSEN-1 and PSEN-2 by BIDs. The only available study on the anti-Alzheimer’s activity of BIDs is the inhibition of AChE/BACE-1 by Belluti et al. (2014) (reference no. 5). Therefore, we aimed to investigate the anti-Alzheimer’s activity through PSEN inhibition. This study provides an insight to the molecular mechanism of inactivating PSEN proteins through blocking their active sites. As this study is an *in silico* investigation, *in vitro* and *in vivo* studies in future could be performed using BIDs aiming for PSEN inhibition.

2. The authors used CHARMM27 force field to get the parameter for protein (Line 126 to 129 in manuscript). However, their studies were based on an obsolete force field, which need a revision with the latest force fields. This can impact the results, weakening some analysis. One should use CHARMM36 instead of 27 (please see [doi.org/10.1021/ct300400x](http://doi.org/10.1021/ct300400x)).

Response: As per the reviewer’s suggestion, the force field used for molecular dynamics simulation was changed from CHARMM27 to CHARMM36. According to the reviewer 2 comments the negative control was need to be added in case of both docking and dynamics simulation processes. Therefore, MD simulations of all the BIDs for both PSEN-1 and PSEN-2 were performed again using CHARMM36 force field.

3. Typically, the two compounds with the best activity and the worst activity are usually selected for comparison in MD study. The authors should add a compound as the negative control.

Response: As per the reviewer’s suggestion, negative controls with worst activity were selected for the comparative study. In case of PSEN-1, BID-19 was selected as the negative control, whereas in case of PSEN-2, BID-20 was selected as the same. The visualization/representation of docking studies, dynamics simulation studies, binding free energy calculations, druglikeliness, and pharmacokinetic analysis were also completed by comparing the BID-16 with negative controls.

4. Table 3 and Table 4 contain the same dataset and should be merged into one table.

Response: As per the reviewer’s suggestion, both Table 3 and Table 4 were merged into one table (now Table 3). The table now contains data of structural differentiation of BIDs as well as their interaction details.

5. One gets the impression that the work was assembled very hastily and without careful control. For instance, there are miscellaneous lines on the Fig.8B, which may be caused by the incorrect modification of the residue number. Also, Fig. 7B and 8B are missing ordinate labels. The units in the pictures should be unified (e.g. Fig7A, 7C, 7D, 7E, 8A, 8C, 8D, 8E). As a suggestion, the authors should use professional drawing software to improve the quality of these pictures.

Response: As per the reviewer’s suggestion, negative controls were added for the MD simulation study. Using CHARMM36 forcefield, all the simulations were re-run for 100 ns. All the trajectories of MD simulations were plotted using XMGRACE. The plots were unified and put in one single picture using InkScape software.
